# The ER stress inducer DMC enhances TRAIL-induced apoptosis in glioblastoma

**DOI:** 10.1186/2193-1801-3-495

**Published:** 2014-09-01

**Authors:** Ingrid A M van Roosmalen, Carlos R Reis, Rita Setroikromo, Saravanan Yuvaraj, Justin V Joseph, Pieter G Tepper, Frank A E Kruyt, Wim J Quax

**Affiliations:** 1 Department of Pharmaceutical Biology, Groningen Research Institute of Pharmacy, University of Groningen, Antonius Deusinglaan 1, Groningen, 9713 AV The Netherlands; 2 Department of Medical Oncology, University of Groningen, University Medical Center Groningen, Hanzeplein 1, Groningen, 9713 GZ The Netherlands; 3 Department of Cell Biology, UT Southwestern Medical Center, Dallas, TX 75390-9039 USA; 4 Department of Pulmonary Medicine, Erasmus Medical Center, Westzeedijk 353, Rotterdam, 3015 AA The Netherlands

**Keywords:** TNF-related apoptosis-inducing ligand (TRAIL), Glioblastoma multiforme (GBM), 2,5-dimethyl-celecoxib (DMC), Endoplasmic reticulum (ER) stress, Apoptosis

## Abstract

Glioblastoma multiforme (GBM) is the most aggressive malignant brain tumour in humans and is highly resistant to current treatment modalities. We have explored the combined treatment of the endoplasmic reticulum (ER) stress-inducing agent 2,5-dimethyl-celecoxib (DMC) and TNF-related apoptosis-inducing ligand (TRAIL WT) or the DR5-specific TRAIL D269H/E195R variant as a potential new strategy to eradicate GBM cells using TRAIL-resistant and -sensitive GBM cells. GBM cell lines were investigated for their sensitivity to TRAIL, DMC and combination of both agents. Cell viability was measured by MTS assay and apoptosis was assessed by Annexin V/PI and acridine orange staining. Caspase activation and protein expression levels were analysed with Western blotting. Death Receptor (DR) cell surface expression levels were quantified by flow cytometry. DR5 expression was increased in U87 cells by ectopic expression using a retroviral plasmid and survivin expression was silenced using specific siRNAs. We demonstrate that A172 expresses mainly DR5 on the cell surface and that these cells show increased sensitivity for the DR5-specific rhTRAIL D269H/E195R variant. In contrast, U87 cells show low DR cell surface levels and is insensitive via both DR4 and DR5. We determined that DMC treatment displays a dose-dependent reduction in cell viability against a number of GBM cells, associated with ER stress induction, as shown by the up-regulation of glucose-regulated protein 78 (GRP78) and CCAAT/-enhancer-binding protein homologous protein (CHOP) in A172 and U87 cells. The dramatic decrease in cell viability is not accompanied by a correspondent increase in Annexin V/PI or caspase activation typically seen in apoptotic or/and necrotic cells within 24h of treatment. Although DMC did not affect DR5 expression in the GBM cells, it increased TRAIL-induced caspase-8 activation in both TRAIL-sensitive and -resistant cells, indicating that DMC potentiates initiator caspase activation in these cells. In A172 cells, sub-toxic concentrations of DMC greatly potentiated TRAIL-induced apoptosis. Furthermore, DMC strongly reduced survivin expression in A172 and U87 cells and silencing of this anti-apoptotic protein partially sensitized cells to TRAIL-induced apoptosis. Our findings corroborate that DMC is a promising agent against GBM, and uncovers a potential synergistic cooperation with TRAIL in this highly malignant cancer.

**Electronic supplementary material:**

The online version of this article (doi:10.1186/2193-1801-3-495) contains supplementary material, which is available to authorized users.

## Background

Glioblastoma multiforme (GBM) has been classified as a WHO grade IV glioma. This type of tumour is the most common and aggressive of the glial tumours. Despite the available treatment modalities, consisting of surgical resection followed by a combination of radiation and chemotherapy, the median survival rate ranges from 12–15 months (Stupp et al. 2005). To improve the prognosis of GBM patients, novel therapies are therefore required.

TNF-related apoptosis-inducing ligand (TRAIL), a member of the TNF superfamily, has previously been shown to be a promising anti-cancer therapeutic agent (Wiley et al. 1995; Pitti et al. 1996) for its ability to induce apoptosis in a variety of tumour cells, without affecting normal cells (Ashkenazi et al. 1999; Lawrence et al. 2001). Trimeric recombinant human TRAIL (rhTRAIL) is able to induce apoptosis upon binding to either death receptor 4 (DR4, TRAIL-R1) or death receptor 5 (DR5, TRAIL-R2). TRAIL receptor-specific apoptosis-inducing variants have previously been generated, displaying enhanced affinity for either DR4 or DR5, resulting in more potent apoptosis induction in several tumour cells (van der Sloot et al. 2006; Reis et al. 2010). Binding of TRAIL to its cognate death receptors triggers the establishment of the death-inducing signalling complex (DISC), composed of FAS-associated death domain (FADD) and pro-caspase-8. Upon DISC formation, the initiator pro-caspase-8 is processed into active caspase-8, which is able to either directly cleave effector caspases-3 and -7, or cleave Bid into truncated Bid (tBid). The latter results in the release of mitochondrial factors, including cytochrome c, subsequently leading to caspase-9 cleavage and further activation of effector caspases, resulting in irreversible apoptosis (Budihardjo et al. 1999). In general, GBM cells have been found to be highly resistant to TRAIL-induced apoptosis due to a variety of reasons, such as moderate to low expression of DR4 and DR5 (Knight et al. 2001; Kuijlen et al. 2006), the up-regulation of the anti-apoptotic proteins c-Flip, Bcl-2 and survivin (Knight et al. 2001; Kouri et al. 2012; Song et al. 2003; Xie et al. 2006; Fulda et al. 2002), or the down-regulation of critical pro-apoptotic proteins such as caspase-8 and Bak (Knight et al. 2001; Song et al. 2003; Capper et al. 2009; Qi et al. 2011).

Recently, an increasing number of reports have shown the therapeutic relevance of inducers of endoplasmic reticulum (ER) stress in cancer, also as sensitizers for TRAIL-based therapies (Chen et al. 2007; Zhou et al. 2013; Yoon et al. 2013; Martin-Perez et al. 2012; Kim et al. 2011; Tian et al. 2011; Gaiser et al. 2008). ER stress can be triggered by alterations in normal ER function, such as the accumulation of unfolded, misfolded or excessive proteins, imbalances of lipids or glycolipids, or changes in the redox or ionic conditions within the ER lumen (Lee 2001, Boyce & Yuan 2006, Wu & Kaufman 2006). The ER stress inducer 2,5-dimethyl-celecoxib (DMC) is an analogue of the cyclooxygenase-2 (COX-2)-selective non-steroidal anti-inflammatory drug (NSAID) celecoxib. At the molecular level, DMC lacks the COX-2 inhibitory function present in celecoxib, whereas the ER stress-activating potential is enhanced by DMC (Pyrko et al. 2007; Chuang et al. 2008). DMC has also been shown to block cell proliferation in several tumour cell culture models (Chuang et al. 2008; Pyrko et al. 2006) and demonstrated anti-tumorigenic activity *in vivo* (Pyrko et al. 2006). ER stress appears to be initiated within seconds after the addition of DMC to cultured cells, through the inhibition of the sarcoplasmic/ER calcium ATPase (SERCA) (Pyrko et al. 2007; Johnson et al. 2002; Tanaka et al. 2005). Consequently, an ER stress response (ESR) is triggered, which is characterized by the up-regulation of ER molecular chaperones, including the pro-survival regulator glucose-regulated protein 78 (GRP78), therefore facilitating protein folding, translocation of polypeptides across the ER membrane, and the activation of transmembrane ER stress sensors (Li & Lee 2006). Another ER stress indicator is the enhanced expression of the pro-apoptotic CCAAT/-enhancer-binding protein homologous protein (CHOP) (Kim et al. 2006; Gorman et al. 2012; Siegelin 2012; Kardosh et al. 2008), which has been found to up-regulate DR5 expression in several cancer cell types (Chen et al. 2007; Zhou et al. 2013; Yoon et al. 2013; Martin-Perez et al. 2012; Kim et al. 2011; Tian et al. 2011; Lee et al. 2008). ER stress has also been reported to down-regulate anti-apoptotic proteins, including c-Flip (Chen et al. 2007; Zhou et al. 2013; Yoon et al. 2013; Martin-Perez et al. 2012), Bcl-2 (Zhou et al. 2013; Lee et al. 2008; McCullough et al. 2001) and survivin (Zhou et al. 2013; Gaiser et al. 2008). Moreover, prolonged activation of ER stress can lead to the activation of caspase-4 (Pyrko et al. 2007; Kardosh et al. 2008; Hitomi et al. 2004) and -7 (Chuang et al. 2008; Kardosh et al. 2008) resulting in apoptosis.

In this study, we have explored the ability of DMC to enhance TRAIL-induced apoptosis in GBM cells. We demonstrate that A172, but not U87, is sensitive for apoptosis induced by rhTRAIL, and especially for the DR5-specific TRAIL variant D269H/E195R. DMC was able to significantly reduce cell viability of several GBM cell lines. We show that both sub-toxic and toxic doses of DMC significantly enhance TRAIL-induced apoptosis in A172 cells. Taken together, DMC in combination with rhTRAIL appears to be a promising therapeutic approach for the treatment of a subset of GBM cells.

## Results

### A172 but not U87 cells are sensitive to TRAIL-induced apoptosis primarily via DR5

**Figure 1:**
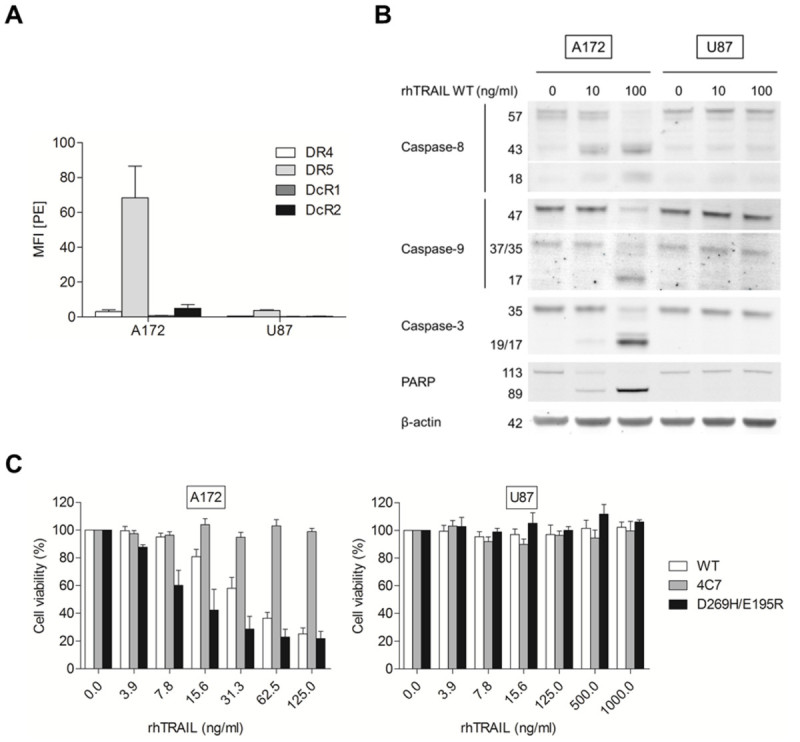
**A172 cells are sensitive to TRAIL-induced apoptosis in a dose-dependent manner. (A)** Cell surface expression of the various TRAIL receptors was determined on A172 and U87 cells using flow cytometry analysis and expressed as the Mean Fluorescence Intensity (MFI) ratio. **(B)** Viability of A172 and U87 cells was assessed after treatment with various concentrations (0–1000 ng/ml) of rhTRAIL WT, 4C7 or D269H/E195R for 24h as measured by MTS assays. **(C)** Western blot analysis of A172 and U87 cells treated with 0, 10 or 100 ng/ml rhTRAIL WT for 5h or 24h, respectively. β-actin serves as a loading control. Error bars represent S.E.M. of three independent experiments.

Analysis of receptor expression by flow cytometry revealed distinct differences in TRAIL receptor membrane expression levels of A172 and U87 cells (Figure 1A). While A172 cells express high levels of DR5, U87 cells showed significantly lower levels of surface DR5. DR4 expression was found to be low in A172 and it was undetectable in U87. Low decoy receptor expression was also detected in A172 cells and absent in U87 cells. Since both A172 and U87 cells show distinct expression profiles of DR5 on the membrane and low or absent expression of DR4, these cells were exposed to different concentrations of rhTRAIL WT, and the previously described DR4-selective variant (rhTRAIL 4C7) (Reis et al. 2010) and DR5-selective variant (rhTRAIL D269H/E195R) (van der Sloot et al. 2006). A172 cells showed a clear dose dependent sensitivity to rhTRAIL, when treated with either rhTRAIL WT or rhTRAIL D269H/E195R, but was completely resistant to rhTRAIL 4C7. The mutant D269H/E195R was more effective in reducing cell viability in A172 when compared to rhTRAIL WT. In contrast, U87 cells were highly resistant to all rhTRAIL ligands (Figure 1B). Afterwards, Western blot analysis was used to detect cleavage of caspases and PARP after treatment with 10 (low) or 100 (moderately high) ng/ml rhTRAIL WT. Since A172 cells proved to be TRAIL-sensitive these cells were exposed to rhTRAIL WT for 5h in order not to lose the cells due to massive cell death. The TRAIL-resistant U87 cells were treated for 24h. Accordingly, rhTRAIL WT exposure resulted in clear cleavage of caspase-8, -9, -3 and PARP in A172 cells, whereas in U87 cells, even upon treatment with 100 ng/ml rhTRAIL WT, no activation of these apoptotic-related proteins was observed (Figure 1C). We then tested if the differential TRAIL-sensitivities observed in A172 and U87 could be explained by the differences at the level of surface DR5 expression, with low levels of death receptor expression in U87 cells causing the insensitivity to both rhTRAIL WT and rhTRAIL D269H/E195R.

### Ectopic overexpression of DR5 in U87 cells does not enhance TRAIL sensitivity

**Figure 2:**
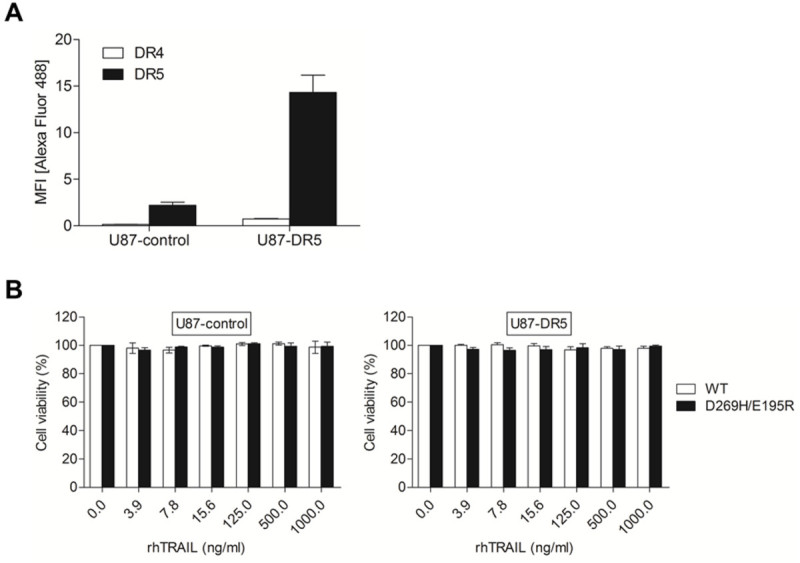
**DR5 overexpression does not enhance TRAIL-induced apoptosis in U87 cells. (A)** Flow cytometry analysis was performed on U87-control and U87-DR5 cells to measure the cell surface expression of TRAIL receptors DR4 and DR5, expressed as the Mean Fluorescence Intensity (MFI) ratio. A172 cells were used as a control. **(B)** Cell viability was assessed after 24h treatment with 0–1000 ng/ml rhTRAIL WT or D269H/E195R using MTS assays. Presented data are representative for three independent experiments and mean cell viability levels ± S.E.M. are shown.

To investigate whether enhancement of DR5 expression could trigger TRAIL-induced apoptosis, U87 cells were transduced with either an empty retroviral vector (U87-control) or a vector expressing DR5 (U87-DR5). This vector contains the gene for tdTomato, allowing for the isolation of transduced tdTomato-positive cells by FACS sorting. DR5 expression levels were determined by flow cytometry using TRAIL receptor-specific antibodies labelled with the Alexa Fluor 488 dye, since PE-labelled antibodies would result in overlapping emission spectra with tdTomato (Figure 2A). Notably, a large shift in mean fluorescence intensity (MFI) levels was measured when Alexa Fluor 488 was used compared to previous results with the PE dye. Although the elevated surface levels of DR5 in U87-DR5 cells is similar to the DR5 expression level observed in A172 cells, U87-DR5 cells did not show enhanced sensitivity to rhTRAIL WT or rhTRAIL D269H/E195R even at high concentrations of rhTRAIL (up to 1000 ng/ml) (Figure 2B). These results indicate that DR5 overexpression *per se* is not sufficient to sensitize U87 cells to TRAIL-induced apoptosis.

### DMC decreases cell viability of GBM cells and enhances TRAIL-induced apoptosis in A172 cells

**Figure 3:**
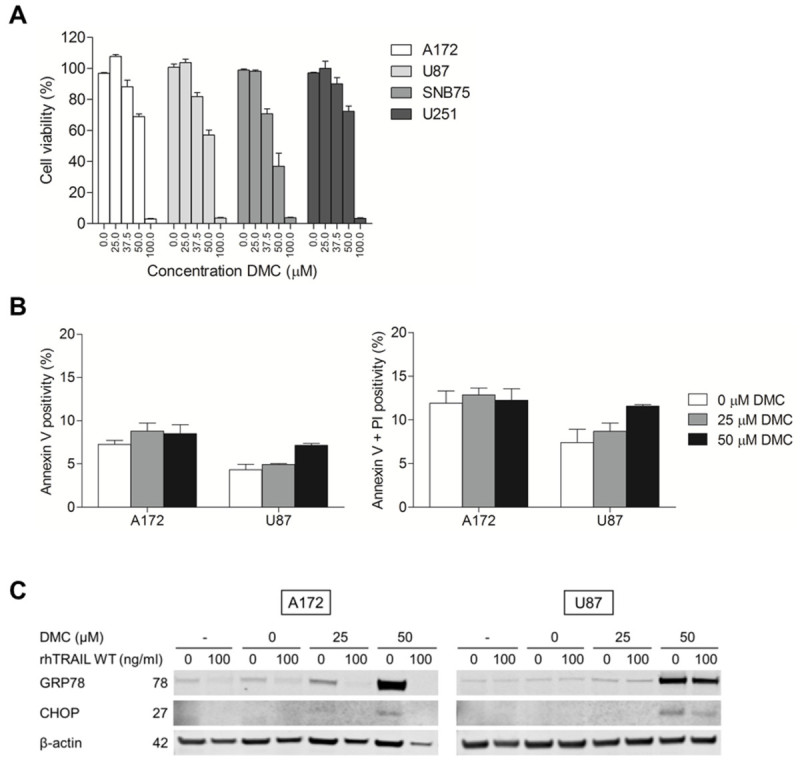
**DMC reduces cell viability and induces ER stress in GBM cell lines. (A)** Cell viability was assessed in a panel of GBM cell lines after 24h exposure to 0–100 μM of DMC using a MTS assay. Error bars represent S.E.M. of three independent experiments. **(B)** A172 and U87 cells were treated with 0, 25 or 50 μM DMC for 24h after which apoptosis induction was determined using Annexin V/PI using flow cytometry. **(C)** Treatment of A172 and U87 cells with 0, 25 or 50 μM DMC for 24h resulted in a dose-dependent up-regulation of CHOP and GRP78 proteins levels as showed by Western blotting. β-actin serves as a loading control. Presented data are representative for at least three independent experiments and mean cell viability levels ± S.E.M. are shown.

Next, we examined the potential of the ER stress inducer DMC alone and in combination with TRAIL across several GBM cell lines. We started by determining the effect of DMC on the cell viability of GBM cells. As shown in Figure 3A, DMC was able to efficiently promote reduction in cell viability as measured by MTS in all the GBM cell lines studied. SNB75 cells showed the highest sensitivity to DMC, whereas U251, A172 and U87 cells were almost equally sensitive to DMC (Figure 3A).

**Figure 4:**
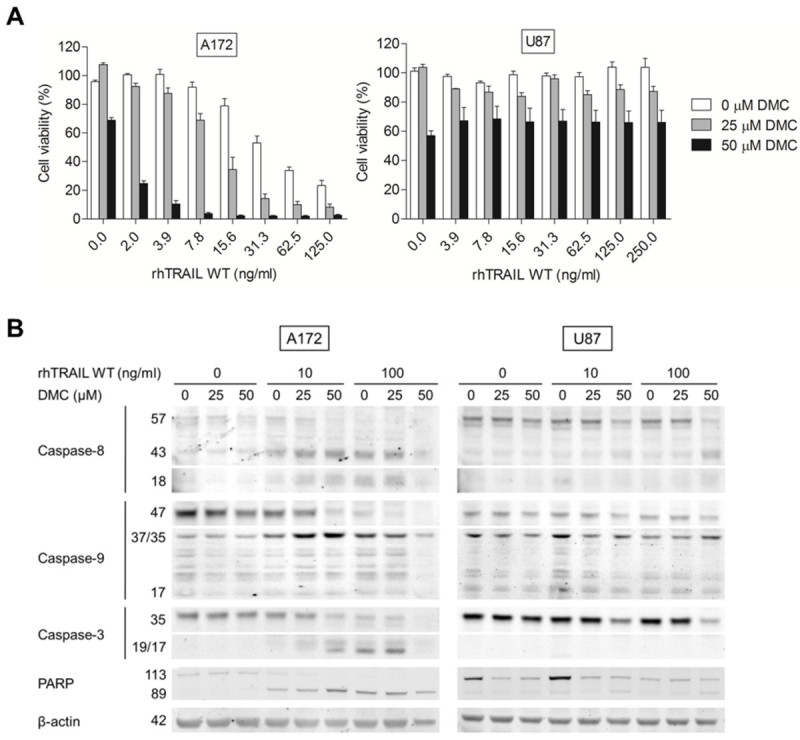
**Increased TRAIL sensitivity by DMC is caspase-dependent. (A)** Cell viability was assessed after 24h co-treatment with rhTRAIL WT (0–250 ng/ml) and 0, 25 or 50 μM DMC using a MTS assay. **(B)** Western blot analysis of A172 (5h) and U87 (24h) cells treated with either rhTRAIL WT (0, 10 or 100 ng/ml) and/or DMC (0, 25 or 50 μM) for caspase-8, -9, -3 and PARP. β-actin served as a loading control.

To evaluate if the DMC effects observed in these cells were correlated with the induction of apoptosis or necrosis, A172 and U87 cells were treated with either a sub-toxic (25 μM) or a moderate cytotoxic (50 μM) dose of DMC for 24h and stained with Annexin V/PI, with Annexin V positive cells representing the apoptotic fraction. Figure 3B shows that the addition of a high concentration of DMC alone resulted in a minor increase in Annexin V positivity. In addition, after DMC treatment the level of Annexin^-^/PI^+^ cells were comparable to untreated cells. Together with the results obtained using MTS assays, these results suggest that treating cells with concentrations up to 50 μM DMC did not result in apoptosis or necrosis in these cells, contrasting with the significant reduction seen when using the MTS assay. In order to assess if DMC induced ER stress, expression levels of GRP78 and CHOP, two established markers of ER stress, were examined. Consistent with the previously described role of DMC in ER-stress induction, Western blot analysis showed a clear up-regulation of GRP78 and CHOP upon treatment with 50 μM of DMC, (Figure 3C). Next, the effect of combined exposure to rhTRAIL WT and 0, 25 or 50 μM of DMC was examined in these differentially TRAIL-responsive GBM cell lines. In this assay, A172 cells treated for 24h with 125 ng/mL rhTRAIL without DMC showed already ~75% reduction in cell viability (IC_50_: ~36 ng/ml), which was strongly enhanced after combination with DMC (25 μM: ~10%, IC_50_: ~12 ng/ml and 50 μM: ~2%, IC_50_: ~1 ng/ml) (Figure 4A). Notably, the cell viability of A172 cells was reduced with approximately 86% using only 31.3 ng/ml of rhTRAIL WT in combination with 25 μM of DMC, when compared to TRAIL WT in combination with 0 μM DMC, where approximately 47% reduction in cell viability could be attained. Therefore, TRAIL sensitivity can be enhanced in A172 cells by co-treatment with DMC. U87 cells remained TRAIL-resistant (0 μM: ~99%), and although the single treatment with DMC was able to lower the cell viability of U87 cells (25 μM: ~87%; 50 μM: 66%), no additive or synergistic effects could be found in combination with rhTRAIL WT (Figure 4A). Since DMC is an analogue of celecoxib, we further tested the sensitivity of these cell lines to celecoxib treatment. A172 and U87 showed similar IC_50_ values of approximately 88 μM for both A172 and U87 (Additional file 1: Figure S1). Next, DMC or celecoxib were tested side-by-side in combination with rhTRAIL WT. Interestingly, the combination of TRAIL/DMC (IC_50_: ~57 μM) was shown to be more effective than TRAIL/celecoxib (IC_50_: ~88 μM) (Additional file 2: Figure S2). Since DMC is a more potent ER stress inducer than celecoxib and does not target COX-2 (Pyrko et al. 2007; Chuang et al. 2008), these results suggest that ER stress activation is closely related to the reduction of cell viability, and subsequently, the enhancement of TRAIL-induced cytotoxicity in these cells.

**Figure 5:**
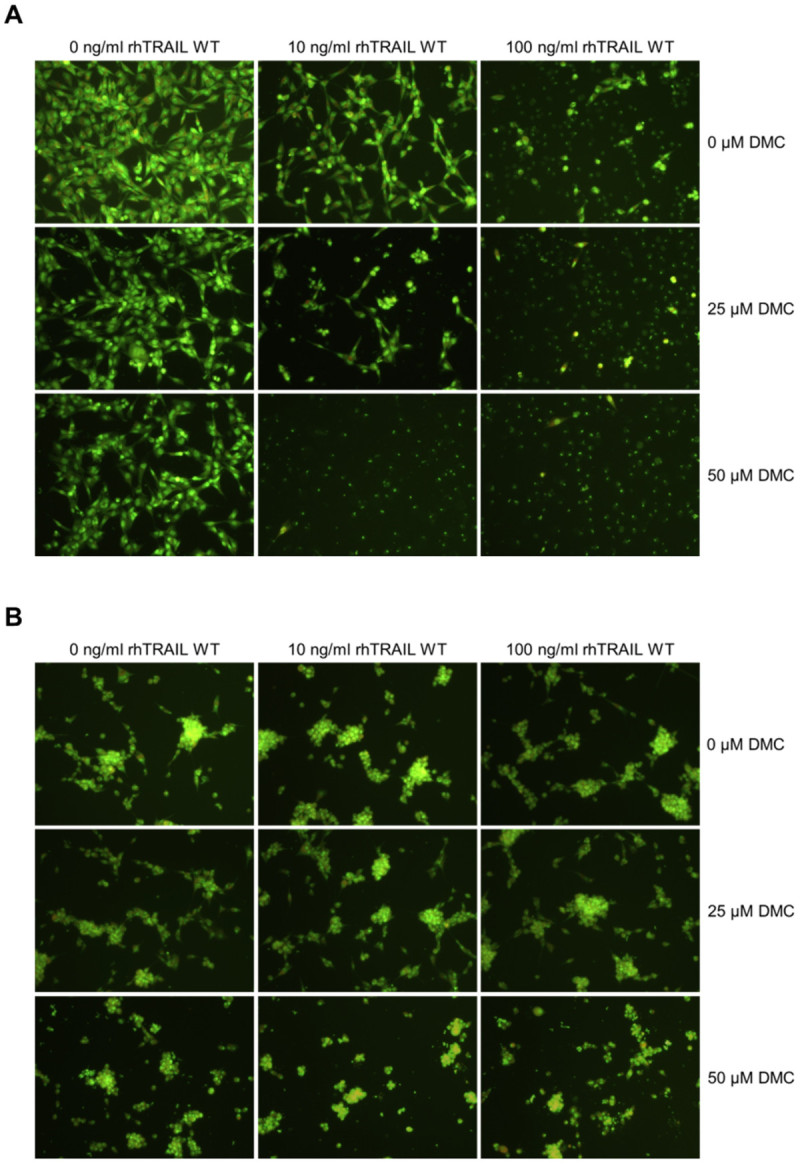
**DMC reduces proliferation in GBM cells.** Acridine orange staining was used to visualize apoptosis in A172 **(A)** and U87 **(B)**. Cells were treated with 0, 10 or 100 ng/ml rhTRAIL WT and 0, 25 or 50 μM DMC. After 24h, cells were stained using acridine orange dye.

To confirm that the combinatorial effects described above were correlated with enhanced apoptosis activity, the effect of DMC on TRAIL-induced caspase cleavage was further analysed. Figure 4B shows that after 5h treatment, A172 cells display a significant activation of the caspases-8, -9, -3 and PARP cleavage when exposed to rhTRAIL WT alone, and enhanced cleavage when combined with DMC. Treatment with 100 ng/ml of rhTRAIL WT and 50 μM DMC resulted in massive cell death in A172 cells. Notably, DMC alone did not induce any activation of downstream apoptosis-related proteins, as indicated by a lack of caspase activation or PARP cleavage, and in agreement with our earlier observations using Annexin V staining assays (Figure 3B). Furthermore, acridine orange staining of DMC-treated cells showed no evidence of nuclear condensation and nuclear/cellular fragmentation, as shown after rhTRAIL WT treatment in A172 cells, but it does appear that DMC reduces cell proliferation, especially in A172 cells (Figure 5). Notably, in U87 cells the combined treatment of rhTRAIL WT with 50 μM DMC led to activation of the initiator pro-caspase-8 but no subsequent pro-caspase-9, -3 or PARP cleavage. Treatment of U87 cells with both TRAIL WT and DMC resulted in the down-regulation of pro-caspase-3 in this cell line (Figure 4B). Finally, the effect of combined exposure to rhTRAIL WT and DMC was also examined in U87-control and U87-DR5 cells, with no significant effect of DMC on TRAIL-sensitivity being observed for these cell lines (Additional file 3: Figure S3).

### DMC down-regulates the anti-apoptotic survivin in A172 and U87 cells

**Figure 6:**
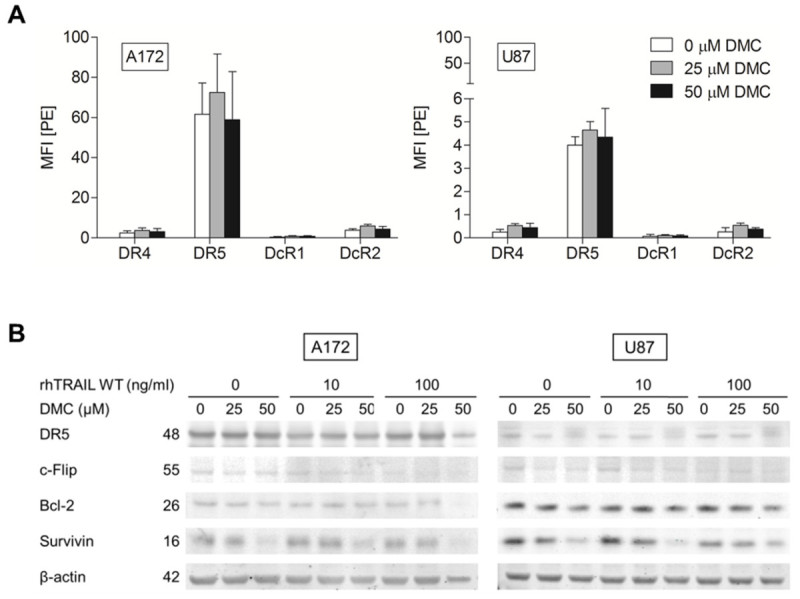
**DMC mainly down-regulates the anti-apoptotic protein survivin. (A)** Flow cytometry analysis was performed after 24h of 0, 25 or 50 μM DMC treatment to measure the cell surface expression of TRAIL receptors in A172 and U87 cells, expressed as the Mean Fluorescence Intensity (MFI) ratio. Error bars represent S.E.M. of three independent experiments. **(B)** Western blot analysis of A172 (5h) and U87 (24h) cells treated with either rhTRAIL WT (0, 10 or 100 ng/ml) and/or DMC (0, 25 or 50 μM). C-Flip, Bcl-2 and survivin protein expression levels were tested. β-actin served as a loading control. Presented data are representative for at least two independent experiments.

Previous studies have reported that ER stress can stimulate apoptosis induction not only by up-regulating pro-apoptotic proteins, such as DR5, but also by down-regulating anti-apoptotic proteins, such as c-Flip (Chen et al. 2007; Zhou et al. 2013; Yoon et al. 2013; Martin-Perez et al. 2012), Bcl-2 (Zhou et al. 2013; Lee et al. 2008; McCullough et al. 2001) and survivin (Zhou et al. 2013; Gaiser et al. 2008). Treatment with sub-toxic or toxic concentrations of DMC did not affect the level of TRAIL receptors on the surface of both A172 and U87 cells (Figure 6A). Interestingly, Western Blot analysis showed that DMC treatment resulted in a significant down-regulation of survivin levels in both cell lines (Figure 6B). Furthermore, c-Flip expression levels were slightly down-regulated in U87 when treating these cells with DMC. Finally, DMC treatment did not increase the expression of DR5 or reduce the expression of the anti-apoptotic protein Bcl-2.

### Enhancement of TRAIL sensitivity in A172 cells by survivin siRNA

**Figure 7:**
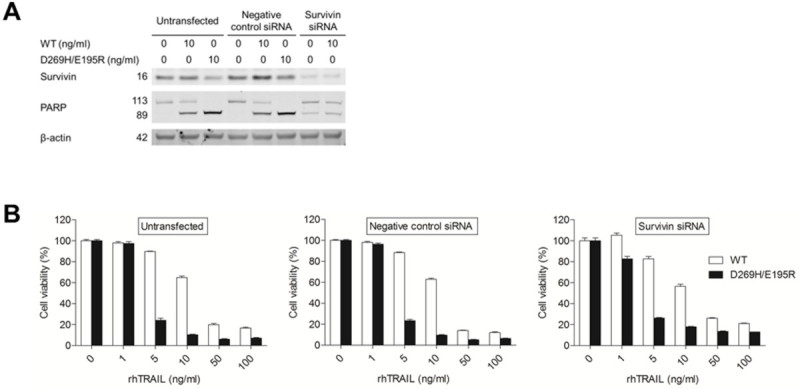
**Sensitivity of A172 to TRAIL-induced apoptosis is slightly enhanced by down-regulation of survivin. (A)** Western blot analysis of survivin siRNA transfected A172 cells treated with or without 10 ng/ml rhTRAIL WT or rhTRAIL D269H/E195R for 5h. Lysates were tested for survivin down-regulation and apoptosis induction by PARP cleavage. β-actin served as a loading control. **(B)** A172 cells transfected with survivin siRNA were treated with 0–100 ng/ml rhTRAIL WT or rhTRAIL D269H/E195R. Cell viability was assessed after 24h using MTS assay. The graphs are corrected for control or survivin knockdown induced cytotoxicity. Presented data are representative for at least three independent experiments and mean cell viability levels ± S.E.M. are shown.

In order to determine if down-regulation of survivin by DMC could explain the increase in TRAIL sensitivity observed in A172 cells, its expression was selectively silenced using a siRNA approach. Figure 7A shows efficient down-regulation of survivin upon siRNA transfection, which resulted in apoptosis activation as indicated by PARP cleavage. Interestingly, the addition of only 10 ng/ml rhTRAIL WT enhanced PARP cleavage in survivin knockdown cells (Figure 7A), whereas the addition of D269H/E195R resulted in massive cell death, such that an insufficient amount of lysate could be prepared. Finally, analysis of cell viability upon TRAIL treatment in non-transfected, control-siRNA and survivin-siRNA treated cells indicate that the down-regulation of survivin slightly increased rhTRAIL WT- and rhTRAIL D269H/E195R-mediated apoptosis in A172 at low concentrations, when compared to both untransfected and control-siRNA treated cells (Figure 7B).

## Discussion

TRAIL is a promising anticancer therapeutic agent that is able to induce apoptosis in various tumour cells while leaving normal cells unharmed (Ashkenazi et al. 1999; Lawrence et al. 2001). Currently, many GBM cells show resistance to TRAIL-induced apoptosis due to various reasons, including low expression of DR4 and DR5 (Knight et al. 2001; Kuijlen et al. 2006), the up-regulation of anti-apoptotic proteins such as c-Flip, Bcl-2 and survivin (Knight et al. 2001; Kouri et al. 2012; Song et al. 2003; Xie et al. 2006; Fulda et al. 2002), or the down-regulation of critical pro-apoptotic proteins such as caspase-8 and Bak (Knight et al. 2001; Song et al. 2003; Capper et al. 2009; Qi et al. 2011). Recently, the use of ER stress inducers, like DMC, have raised great interest as potential anti-cancer agents and sensitizers for TRAIL-based therapies, since ER stress was reported to down-regulate important anti-apoptotic proteins, including c-Flip (Chen et al. 2007; Zhou et al. 2013; Yoon et al. 2013; Martin-Perez et al. 2012), Bcl-2 (Zhou et al. 2013; Lee et al. 2008; McCullough et al. 2001) and survivin (Zhou et al. 2013; Gaiser et al. 2008). Here, we investigated the cell death induction properties of DMC in a panel of GBM cells, as well as the potential of DMC in enhancing TRAIL-induced apoptosis in TRAIL-resistant and -sensitive GBM cells.

We find that A172 and U87 express predominantly DR5 but not DR4 on their surface, with different levels of expression observed between cell lines (Figure 1A). The DR5-high expressing A172 cells were especially sensitive to apoptosis induced by the rhTRAIL variant D269H/E195R, while U87 cells were resistant to apoptosis induction via both DR4 and DR5 (Figure 1B). Interestingly, the ectopic overexpression of DR5 in U87 cells does not lead to enhancement of TRAIL sensitivity, indicating that lowered surface death receptor levels is not the main reason for TRAIL resistance in this cell line, and further suggesting that downstream processes prevent the activation of the TRAIL-apoptosis cascade (Figure 2A-B).

We further studied the impact of ER stress inducer DMC, alone and in the context of TRAIL-mediated apoptosis. We demonstrate that DMC is able to efficiently reduce the cell viability of a panel of GBM cells and induce ER stress as seen by the up-regulation of the ER stress proteins GRP78 and CHOP in A172 and U87 cells (Figure 3). Although A172 and U87 show a clear dose-dependent reduction in cell viability upon treatment with DMC, this is not accompanied by an enhancement of Annexin V or PI staining in these two cell lines (Figure 3B), the activation of caspases (Figure 4B) or the appearance of nuclear condensation (Figure 5), indicating that DMC did not trigger apoptosis or necrosis in these GBM cells after 24h incubation using the mentioned concentrations. However, looking at Figure 5, it appears that DMC is reducing proliferation rates, especially in A172 cells.

The combination of rhTRAIL WT or the DR5-specific variant with middle or high concentrations of DMC resulted in a significant increase in apoptosis induction in A172, leading to nearly complete apoptosis induction at very low concentrations of the ligand rhTRAIL when combined with DMC (Figure 4 and 5). Although the up-regulation of DR5 (Chen et al. 2007; Zhou et al. 2013; Yoon et al. 2013; Martin-Perez et al. 2012; Kim et al. 2011; Tian et al. 2011; Lee et al. 2008) or down-regulation of c-Flip (Chen et al. 2007; Zhou et al. 2013; Yoon et al. 2013; Martin-Perez et al. 2012), Bcl-2 (Zhou et al. 2013; Lee et al. 2008; McCullough et al. 2001) or survivin (Zhou et al. 2013; Gaiser et al. 2008; Pyrko et al. 2006) have been previously described as molecular mechanisms accountable for the sensitizing effect of ER stress induction (or DMC treatment on TRAIL-induced apoptosis), we only detected down-regulation of survivin in these cell lines upon treatment with DMC (Figure 6). Survivin down-regulation induced apoptosis in A172 cells and only slightly affected rhTRAIL WT and D269H/E195R sensitivity in this cell line (Figure 7). The fact that survivin-mediated knockdown only partially affects the sensitivity of this cell line towards TRAIL-induced apoptosis indicate that other mechanisms, perhaps ER-stress independent, may further contribute to the synergistic effects observed for TRAIL in combination with DMC. Furthermore, the depletion of survivin by siRNA led to the induction of apoptosis in A172, contrasting to the lack of clear apoptosis-inducing activity by the ER stress agent DMC.

Recently, Kardosh *et al.* have assigned the anti-tumour effect of DMC to the inhibition of cell proliferation through the down-regulation of cyclins A and B and the consequent loss of cyclin-dependent kinase activity. In that report, the authors have demonstrated the dose-dependent down-regulation of cell cycle-regulatory proteins in the presence of 0, 25 and 50 μM of DMC or celecoxib (Kardosh et al. 2005). A recent study by Ehrhardt *et al.* described the increased sensitivity to TRAIL-induced apoptosis in cell cycle-arrested tumour cells (Ehrhardt et al. 2013). These findings, combined with our observation that synergistic effects can be observed when using DMC in combination with TRAIL in a subset of GBM cells, give us further insight in the pleiotropic nature of this class of compounds and their potential effects in further enhancing or sensitizing cancer cells to TRAIL-induced apoptosis.

In contrast to the results obtained for A172 cells, DMC was not sufficient to circumvent TRAIL-resistance in U87 and DR5 overexpressing U87 cells, even when both c-Flip and survivin down-regulation was observed after treatment with 50 μM DMC. While the combined treatment of rhTRAIL WT with 50 μM DMC led to a clear activation of pro-caspase-8, subsequent cleavage of pro-caspase-9, -3 and PARP could not be detected. Intriguingly, combination treatment of U87 led to a significant reduction in the expression of pro-caspase-3. Yet, U87 cells remain resistant to TRAIL, likely due to reduced pro-caspase-3 expression levels or other persistent downstream mechanisms of resistance that are in place in this particular cell line. Although several mechanisms of caspase-3 down-regulation have been previously described (Suzuki et al. 2001; Chen et al. 2003; Mica et al. 2004; Tan et al. 2006), it is currently unclear how the combination of TRAIL/DMC results in reduction in pro-caspase-3 expression in this cell line.

## Conclusions

Our data clearly shows DMC might be a potential anti-cancer agent for the treatment of GBM, that can further potentiate TRAIL-induced apoptosis in a subset of GBM. DMC alone appears to effectively decrease the cell viability of a panel of GBM cells. Importantly, reduction of cell viability by 24h treatment with DMC did not lead to apoptosis or necrosis, but seems be due to reduced proliferation. We find that the DMC-mediated down-regulation of survivin partially accounts for the mechanism of TRAIL sensitization in A172 cells. As rhTRAIL warrants high tumour selectivity and sub-toxic concentrations of DMC further sensitize these cancer cells to TRAIL, it will be important to assess how other cells, especially primary cells, respond to such combination modalities. Additionally, the anti-tumorigenic properties of DMC alone and in the context of co-treatment will require further *in vitro* and *in vivo* studies, especially considering how effective cancer-specific apoptosis induction may provide therapeutic opportunities for the treatment of GBM as well as many other cancers.

## Materials and methods

### Cell lines and chemicals

Human glioblastoma cell lines A172, U87, SNB75 and U251 were obtained from the American Type Culture Collection (ATCC). Cells were cultured in DMEM with 4.5 g/L D-glucose (Gibco, Life Technologies) supplemented with 10% foetal calf serum, 100 units/mL penicillin and 100 μg/ml streptomycin in a humidified incubator at 37°C containing 5% CO_2_. 2,5-Dimethyl-celecoxib (DMC) was produced as described earlier (Kardosh et al. 2005; Ahlstrom et al. 2007). Celecoxib (CXB) was purchased from Key Organics. DMC and CXB were both dissolved in DMSO; the control with only solvent (DMSO) showed no toxicity. RhTRAIL wild-type (WT), DR4-specific TRAIL variant 4C7 and DR5-specific TRAIL variant D269H/E195R (amino acids 114–281) were constructed and produced as described earlier (van der Sloot et al. 2006; Reis et al. 2010).

### Cell viability and apoptosis assays

Cell viability was measured using the MTS assay, a colorimetric method in which viable cells reduce 3-(4,5-dimethylthiazol-2-yl)-5-(3-carboxymethoxyphenyl)-2-(4-sulfophenyl)-2H-tetrazolium (MTS) to a formazan product. Cells were seeded in triplicate in 96-well plates at a cell density of 10,000 cells/well. After 24h, cells were treated for 24h with concentrations ranging from 0 to 250 ng/mL of rhTRAIL WT or D269H/E195R and/or 0 to 100 μM DMC in a final volume of 0.15-0.2 mL; reagents and ligands were serially diluted in cell culture medium. Cells were incubated with the MTS reagent according to the manufacturer’s instructions (G3581, Promega). Cell viability was determined by measuring the absorption at 492 nm on a microplate reader (Thermo Labsystems). Apoptosis induction was measured using Annexin V-FITC and propidium iodide (PI) staining and quantified by flow cytometry. Cells were seeded in 6-well plates 24 h prior to treatment. The next day, cells were treated for 24h with 0, 25 or 50 μM DMC. After treatment, cells were harvested and washed with calcium buffer (10.9 μM HEPES, 140 μM NaCl, 2.5 μM CaCl_2_). Cell pellets were resuspended in 60 μL calcium buffer complemented with 5 μL Annexin V-FITC (IQP-120F, IQ Products) and incubated for 20 minutes on ice. Cells were washed and resuspended in 0.5 μg/mL PI (P4170, Sigma Aldrich) diluted in calcium buffer and left on ice until analysis. Cells were analysed using a FACSCalibur flow cytometer (BD).

Visualisation of apoptosis was accomplished using acridine orange. Cells were seeded in triplicate in 96-well plates with 10,000 cells/well 24h prior to treatment. Treatment consisted of rhTRAIL WT (0, 10 or 100 ng/mL), with or without DMC (0, 25 or 50 μM) as indicated. After 24h incubation, cells were stained with 2 μL/well 10x diluted 1 mg/ml acridine orange solution and incubated for 10 minutes. Plates were spun down for 10 minutes at 900 rpm. Fluorescence microscopy was used to determine the presence of apoptotic bodies and/or chromatin condensation.

### Western blotting

GBM cells were seeded in T25 culture flasks at a density of 750,000 cells/flask 24h prior to treatment. For TRAIL-induced apoptosis, A172 cells were treated for 5h and U87 for 24h, since apoptosis is induced quickly in A172 cells. Cells were treated with rhTRAIL WT (0, 10 or 100 ng/mL), with or without DMC (0, 25 or 50 μM). After treatment, cells were harvested and lysed using the M-PER Mammalian Protein Extraction Reagent (PIERCE, Thermo Scientific) with additional Protease Inhibitor Cocktail, EDTA-Free (100x; Thermo Scientific). Protein concentrations were determined using a Bradford assay (Bio-Rad Laboratories). Equal amounts of protein for each sample were loaded per lane on pre-cast 4-12% SDS-PAGE gels (Invitrogen) and transferred onto Immobilon-FL PVDF 0.45 μm membranes (Millipore). Subsequently, the membranes were blocked for 1 h at room temperature in blocking buffer (Rockland). Western Blot membranes were probed overnight at 4°C. The following primary antibodies were used: caspase-3 (9662, 9661), caspase-8 (9746), caspase-9 (9501, 9508), c-Flip (8510), CHOP (2895), PARP (9542) (Cell Signaling), COX-2 (160112, Cayman Chemical), GRP78 (sc-13968, Santa Cruz) and survivin (AF886, R&D Systems). Goat-α-mouse-IRDye (800CW; 926–32210 and 680; #926-32220) or goat-α-mouse-IRDye (800CW; 926–32211 and 680; #926-32221) secondary antibodies (Westburg) were used for detection using a LI-COR Odyssey Infrared Imaging System (Westburg). Membranes were probed with anti-β-actin (0869100, MP Biomedicals) to confirm equal loading.

### TRAIL receptor expression analysis

GBM cells were harvested and washed with standard buffer (PBS/1% BSA). TRAIL receptor cell surface expression was determined using 10 μg/mL TRAIL-R1 (ALX-804-297), TRAIL-R2 (ALX-804-298), TRAIL-R3 (ALX-804-344), TRAIL-R4 (ALX-804-299) (Alexis Biochemicals, Enzo Life Sciences.), DR5-01-1 (EXB-11-461, Exbio) or negative control mouse IgG1 (X0931, DAKO). Cells were incubated with primary antibodies for 1 h. Subsequently, the cells were washed and incubated for 1 h with R-phycoerythrin (PE) conjugated goat anti-mouse antibody (1010–09, Southern Biotech) or Alexa Fluor 488 conjugated goat anti-mouse antibody (A-11001, Invitrogen). Receptor cell surface expression was analysed using a FACSCalibur flow cytometer (BD).

### Retroviral-based DR5 overexpresssion

DR5-TV1 was amplified from a plasmid (kindly provided by MSD) using forward (5′-agatctatggaacaacggggacagaacg-3′) and reverse (5′-cgcgaattcttaggacatggcagagt-3′) primers containing BglII and EcoRI restriction sites. The PCR product was cloned into pMSCV-tdTomato, a retrovirus expression system (kindly provided by prof. J.J. Schuringa). For the packaging of the retroviral particles, 2 × 10^6^ HEK293 cells were plated in 94.0 mm cell culture dishes. The next day, cells were transfected with either pMSCV-tdTomato vector expressing DR5-TV1 or an empty vector, using CaCl_2_. After 24h, the medium containing virus particles was harvested, filtered and added to U87 cells, which were plated the day before at a density of 0.25 × 10^6^ U87 cells in wells of a 6-wells plate. The U87 cells were exposed to the viral particles for 48 h after which the virus was removed and fresh medium was added. Mixed populations of U87-empty or U87-DR5 cells were cultured in DMEM with 4.5 g/L D-glucose (Gibco, Life Technologies) supplemented with 10% foetal calf serum, 100 units/mL penicillin, and 100 μg/ml streptomycin in a humidified incubator at 37°C containing 5% CO_2_. U87 transduced cells, containing constructs encoding tdTomato, were sorted using a fluorescent activated cell sorter.

### RNA interference

A172 cells were seeded in 6-wells plates at a density of 200,000 cells/well. The next day, the subconfluent cultures were incubated with unsupplemented Optimem medium (Gibco, Life Technologies) and transfected with 133 nM survivin (sc-29499, Santa Cruz) or negative control small interfering RNA (siRNA) (SR-CL000-005, Eurogentec) using Oligofectamine Transfection Reagent (12252–011, Invitrogen) according to the manufacturer’s protocol. 24h after siRNA transfection, cells were seeded and treated as indicated for MTS assays (24h treatment) or Western blot analysis (5h treatment).

## Electronic supplementary material

Additional file 1: Figure S1: A172 and U87 cells are less sensitive to celecoxib when compared to DMC. Cell viability was assessed after 24h exposure of A172 (A) or U87 (B) to 0–100 μM of CXB or DMC using a MTS assay. Error bars represent S.E.M. of three independent experiments. (TIFF 538 KB)

Additional file 2: Figure S2: DMC is more potent in reducing cell viability when combined with TRAIL than celecoxib. Cell viability was assessed after 24h co-treatment of A172 (A) or U87 (B) with rhTRAIL WT (0–250 ng/ml) and 0, 25 or 50 μM celecoxib (CXB) or DMC using MTS assays. Error bars represent S.E.M. of three independent experiments. (TIFF 355 KB)

Additional file 3: Figure S3: DR5 overexpression in U87 cells did not enhance sensitivity to TRAIL in combination with DMC. Viability was assessed after 24h co-treatment of U87-control (A) or U87-DR5 (B) with 0–1000 ng/ml rhTRAIL WT and 0, 25 or 50 μM DMC using MTS assays. Presented data are representative for three independent experiments and mean cell viability levels ± S.E.M. are shown. (TIFF 485 KB)

Below are the links to the authors’ original submitted files for images.

Authors’ original file for figure 1

Authors’ original file for figure 2

Authors’ original file for figure 3

Authors’ original file for figure 4

Authors’ original file for figure 5

Authors’ original file for figure 6

Authors’ original file for figure 7

Authors’ original file for figure 8

Authors’ original file for figure 9

Authors’ original file for figure 10

## Abbreviations

DMC: 2,5-dimethyl-celecoxib
TRAIL: TNF-related apoptosis-inducing ligand
GBM: Glioblastoma multiforme
ER stress: Endoplasmic reticulum stress
rhTRAIL (WT): Recombinant human TRAIL (wild type)
DR4/5: Death receptor 4/5
DISC: Death-inducing signalling complex
FADD: FAS-associated death domain
tBid: Truncated Bid
COX-2: Cyclooxygenase-2
NSAID: Non-steroidal anti-inflammatory drug
SERCA: Sarcoplasmic/ER calcium ATPase
ERS: ER stress response
GRP78: Glucose-regulated protein 78
CHOP: CCAAT/-enhancer-binding protein homologous protein
ATCC: American type culture collection
PI: Propidium iodide
M-PER: Mammalian protein extraction reagent
PE: R-phycoerythrin
siRNA: Small interfering RNA
MFI: Mean fluorescence intensity.
