# Erratum to: the ER stress inducer DMC enhances TRAIL-induced apoptosis in glioblastoma

**DOI:** 10.1186/2193-1801-3-738

**Published:** 2014-12-15

**Authors:** Ingrid AM van Roosmalen, Carlos R Reis, Rita Setroikromo, Saravanan Yuvaraj, Justin V Joseph, Pieter G Tepper, Frank AE Kruyt, Wim J Quax

**Affiliations:** Department of Pharmaceutical Biology, Groningen Research Institute of Pharmacy, University of Groningen, Antonius Deusinglaan 1, Groningen, 9713 AV The Netherlands; Department of Medical Oncology, University of Groningen, University Medical Center Groningen, Hanzeplein 1, Groningen, 9713 GZ The Netherlands; Department of Cell Biology, UT Southwestern Medical Center, Dallas, TX 75390-9039 USA; Department of Pulmonary Medicine, Erasmus Medical Center, Westzeedijk 353, Rotterdam, 3015 AA The Netherlands

## Correction

The figure numbering in the HTML version of the original article (van Roosmalen et al. 2014) was listed incorrectly, while the PDF version was correct.

Figure 1 in the HTML version is Figure 2 in PDF

Figure 2 in the HTML version is Figure 3 in PDF

Figure 3 in the HTML version is Figure 4 in PDF

Figure 4 in the HTML version is Figure 5 in PDF

Figure 5 in the HTML version is Figure 6 in PDF

Figure 6 in the HTML version is Figure 7 in PDF

Figure 7 in the HTML version is a duplicate of additional Figure S3.

The publisher would like to apologise for this error.
