# Supplementary figures and images for: The ER stress inducer DMC enhances TRAIL-induced apoptosis in glioblastoma
Source: Springerplus. 2014 Sep 1;3(1):495. doi: 10.1186/2193-1801-3-495 (PMC4554544; doi:10.1186/2193-1801-3-495)

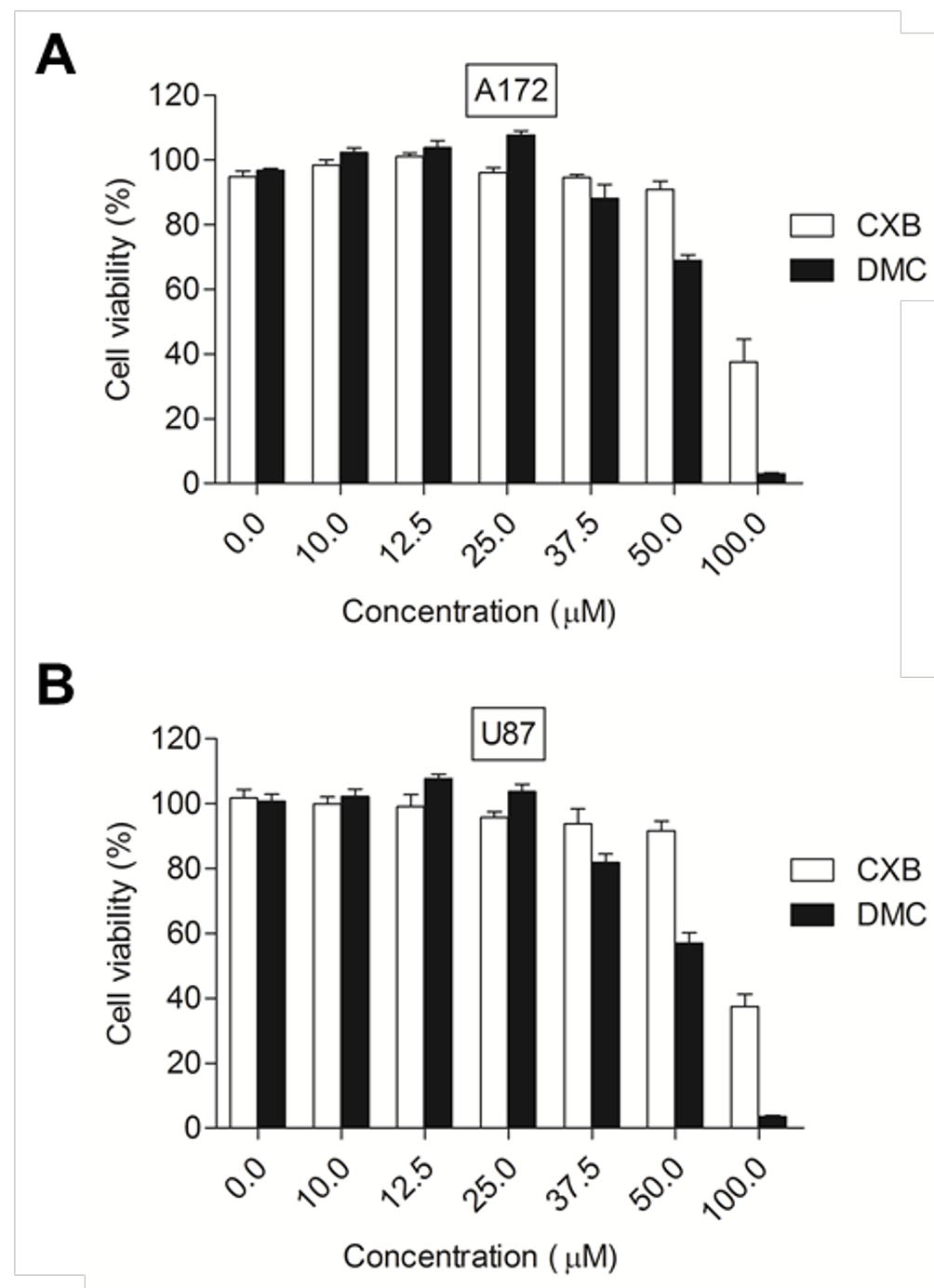

Supplement: Supplementary file 1 — Additional file 1: Figure S1: A172 and U87 cells are less sensitive to celecoxib when compared to DMC. Cell viability was assessed after 24h exposure of A172 (A) or U87 (B) to 0–100 μM of CXB or DMC using a MTS assay. Error bars represent S.E.M. of three independent experiments. (TIFF 538 KB) [file 40064_2014_1207_MOESM1_ESM.tiff]

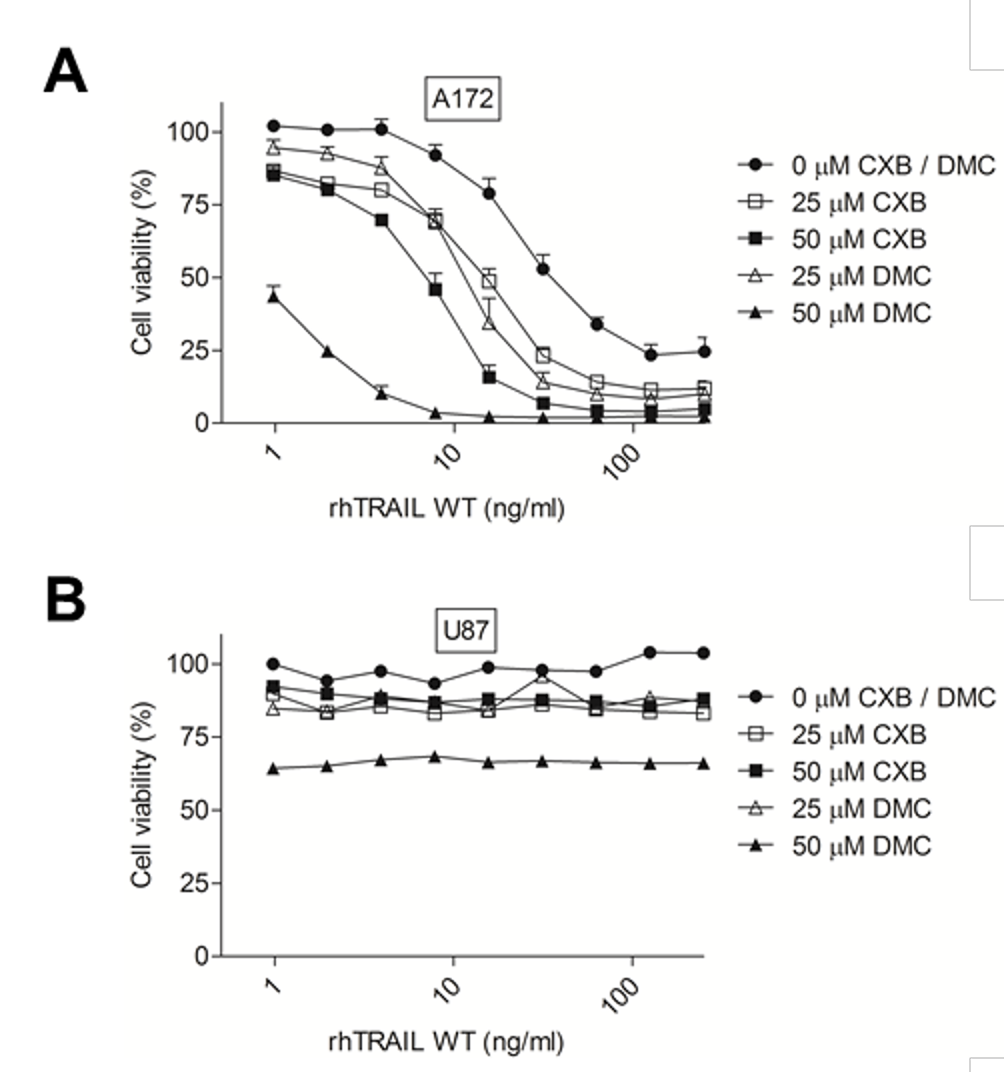

Supplement: Supplementary file 2 — Additional file 2: Figure S2: DMC is more potent in reducing cell viability when combined with TRAIL than celecoxib. Cell viability was assessed after 24h co-treatment of A172 (A) or U87 (B) with rhTRAIL WT (0–250 ng/ml) and 0, 25 or 50 μM celecoxib (CXB) or DMC using MTS assays. Error bars represent S.E.M. of three independent experiments. (TIFF 355 KB) [file 40064_2014_1207_MOESM2_ESM.tiff]

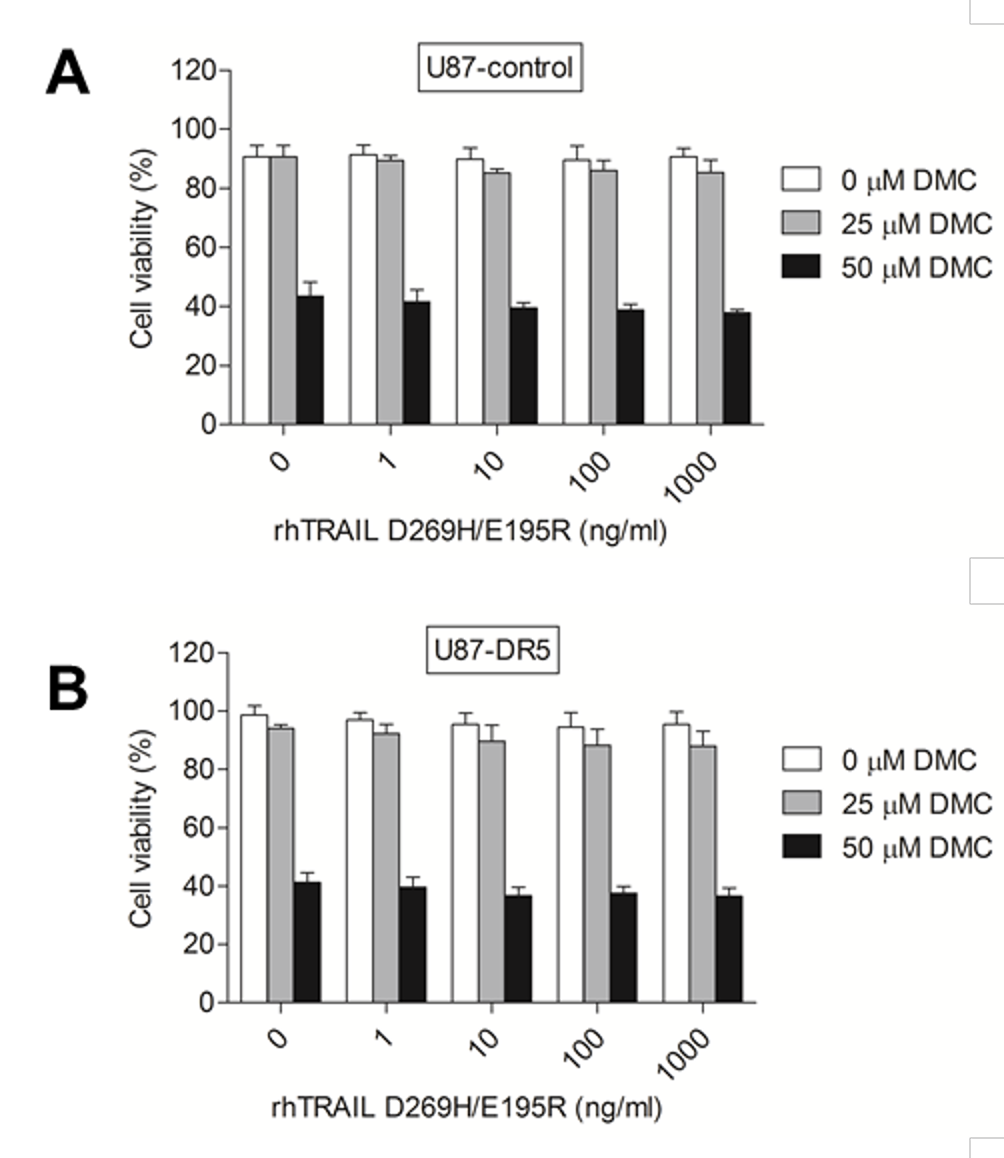

Supplement: Supplementary file 3 — Additional file 3: Figure S3: DR5 overexpression in U87 cells did not enhance sensitivity to TRAIL in combination with DMC. Viability was assessed after 24h co-treatment of U87-control (A) or U87-DR5 (B) with 0–1000 ng/ml rhTRAIL WT and 0, 25 or 50 μM DMC using MTS assays. Presented data are representative for three independent experiments and mean cell viability levels ± S.E.M. are shown. (TIFF 485 KB) [file 40064_2014_1207_MOESM3_ESM.tiff]

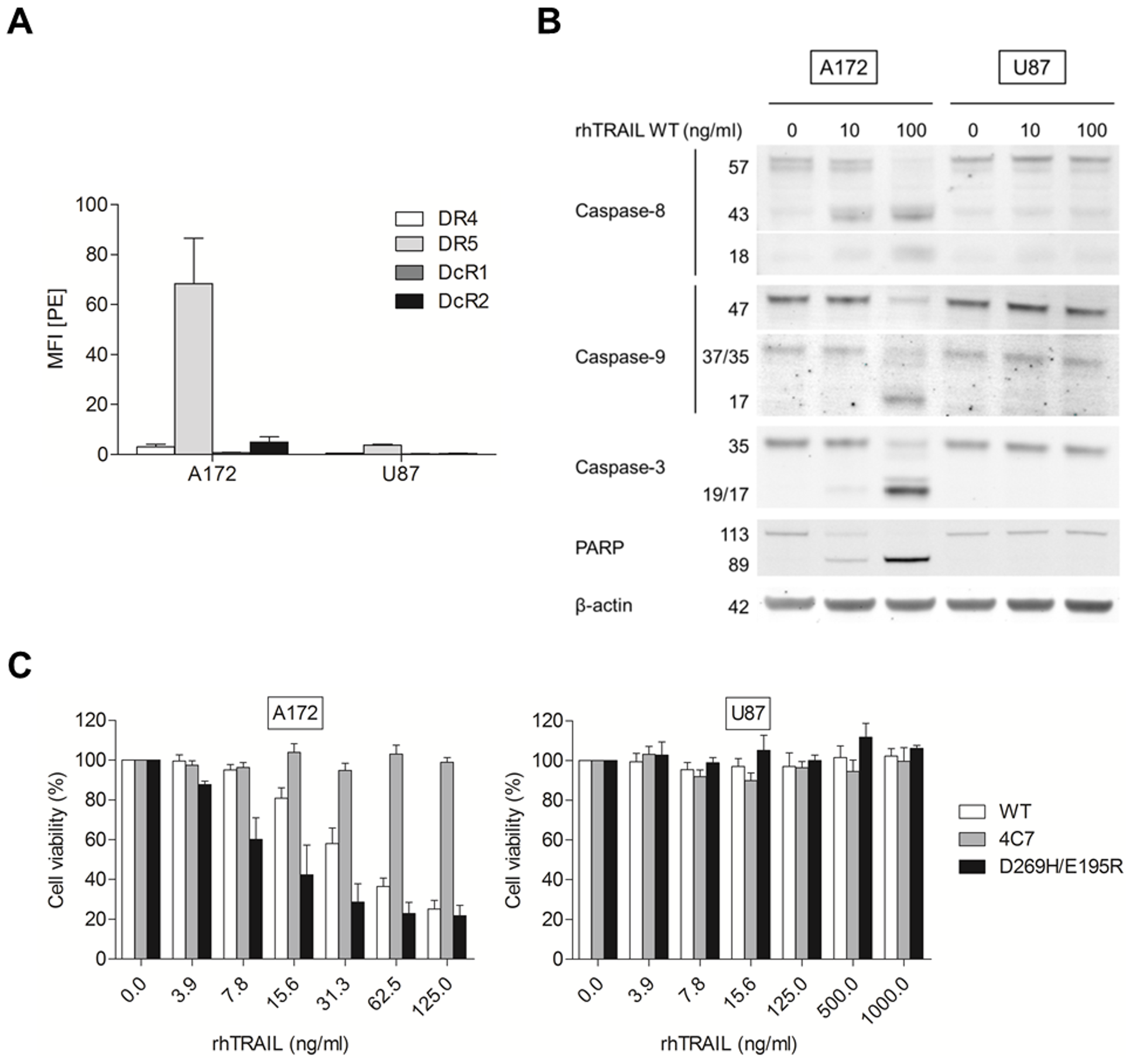

Supplement: Supplementary file 4 — Authors’ original file for figure 1 [file 40064_2014_1207_MOESM4_ESM.tiff]

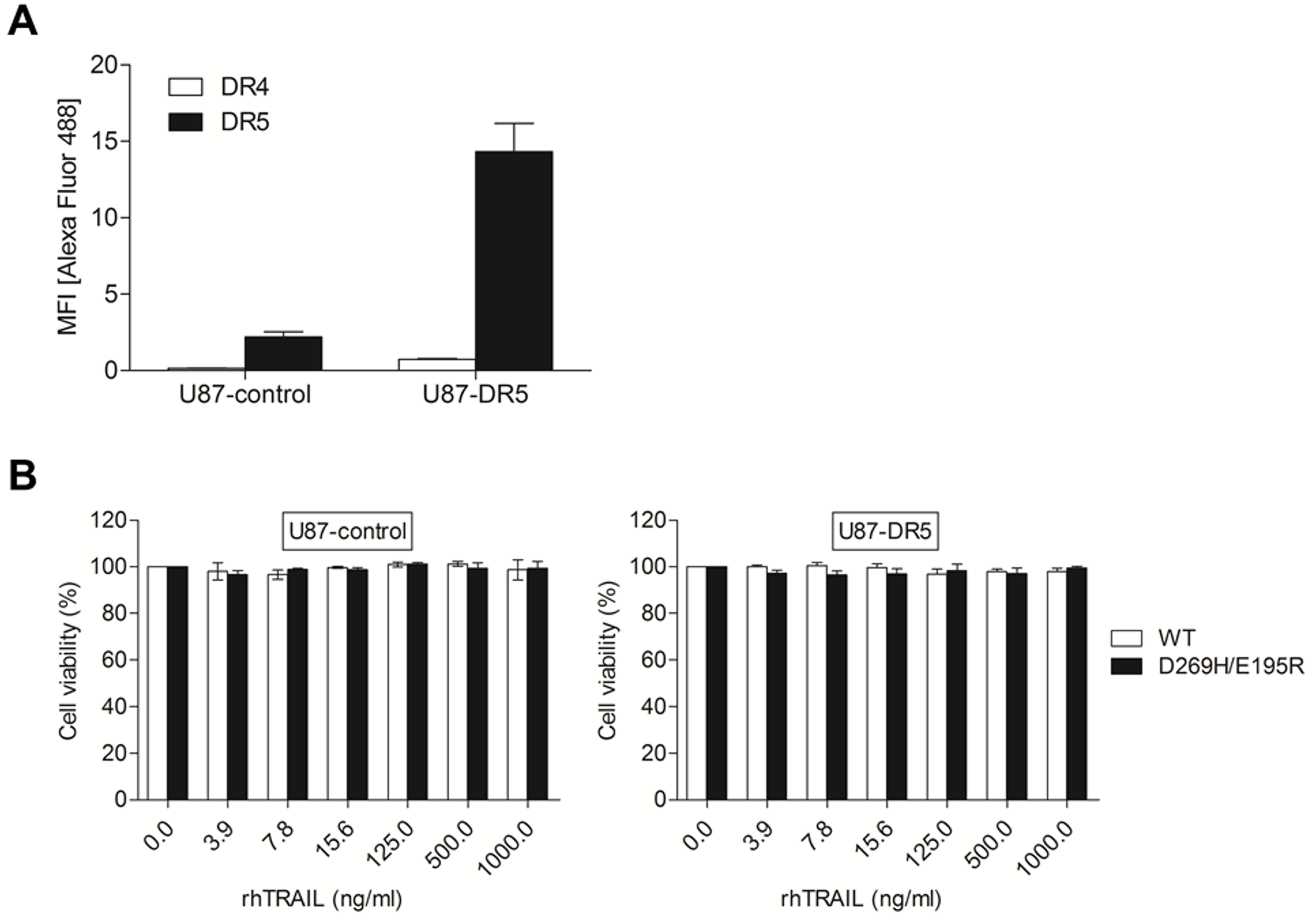

Supplement: Supplementary file 5 — Authors’ original file for figure 2 [file 40064_2014_1207_MOESM5_ESM.tiff]

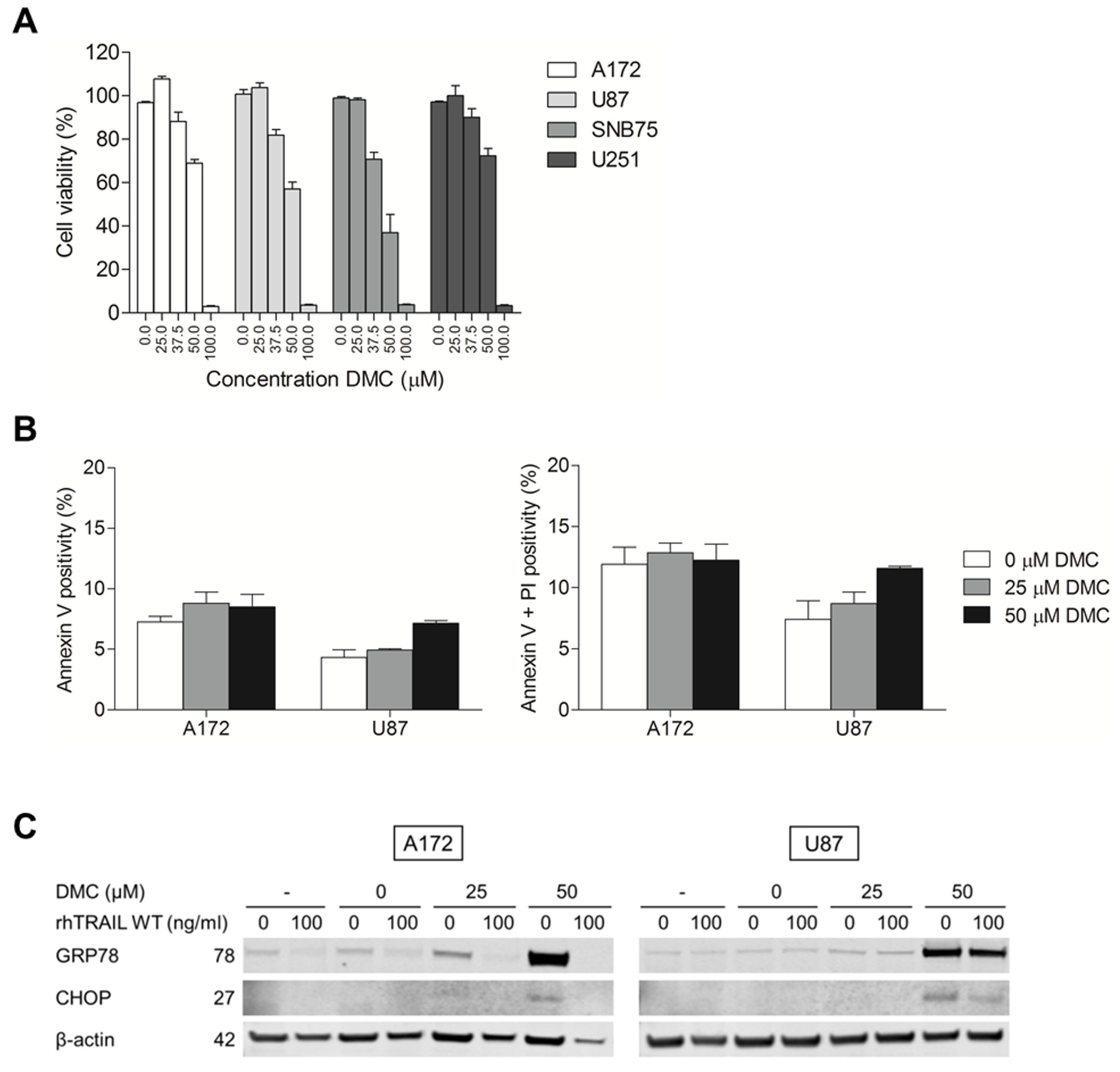

Supplement: Supplementary file 6 — Authors’ original file for figure 3 [file 40064_2014_1207_MOESM6_ESM.tiff]

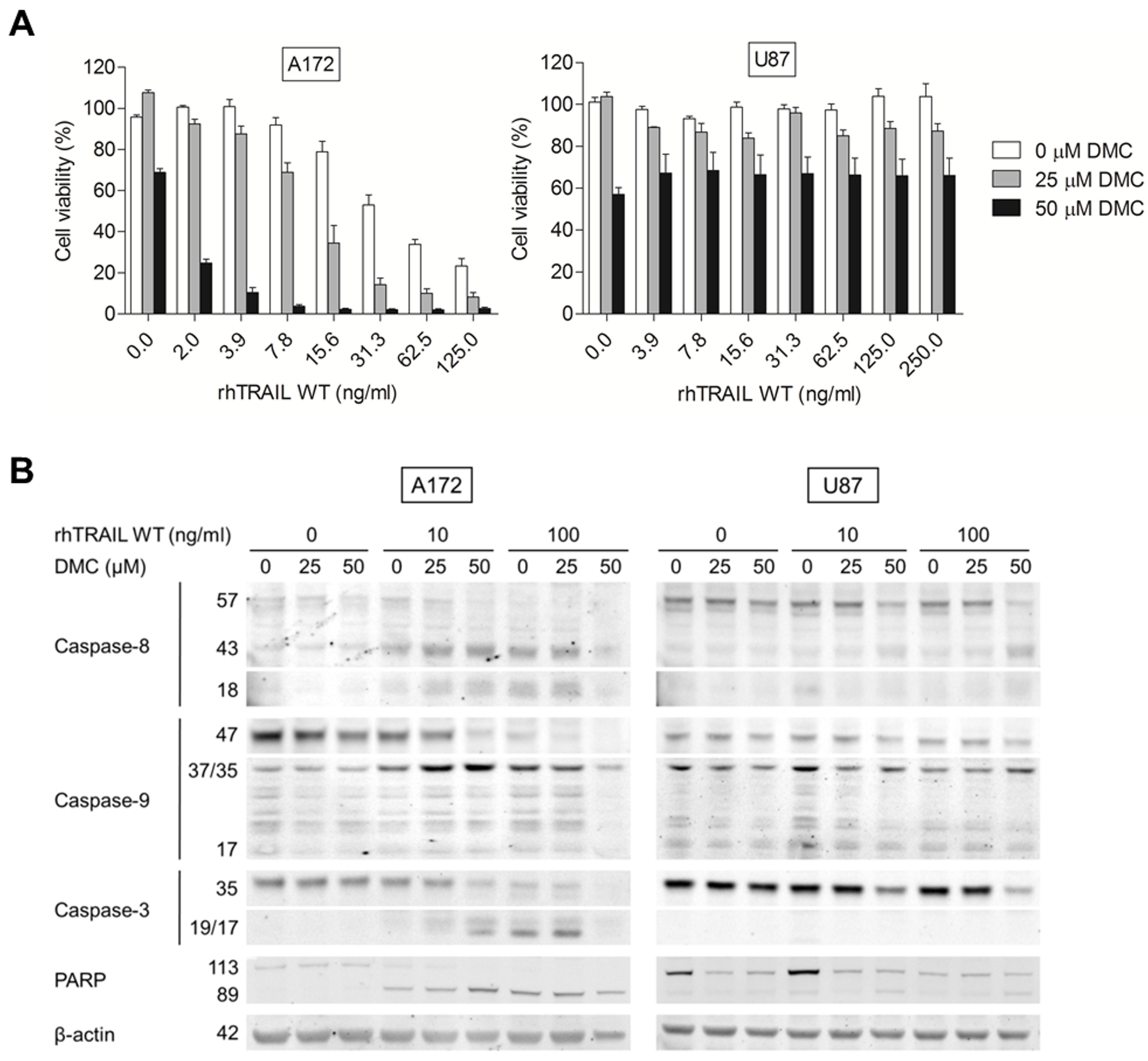

Supplement: Supplementary file 7 — Authors’ original file for figure 4 [file 40064_2014_1207_MOESM7_ESM.tiff]

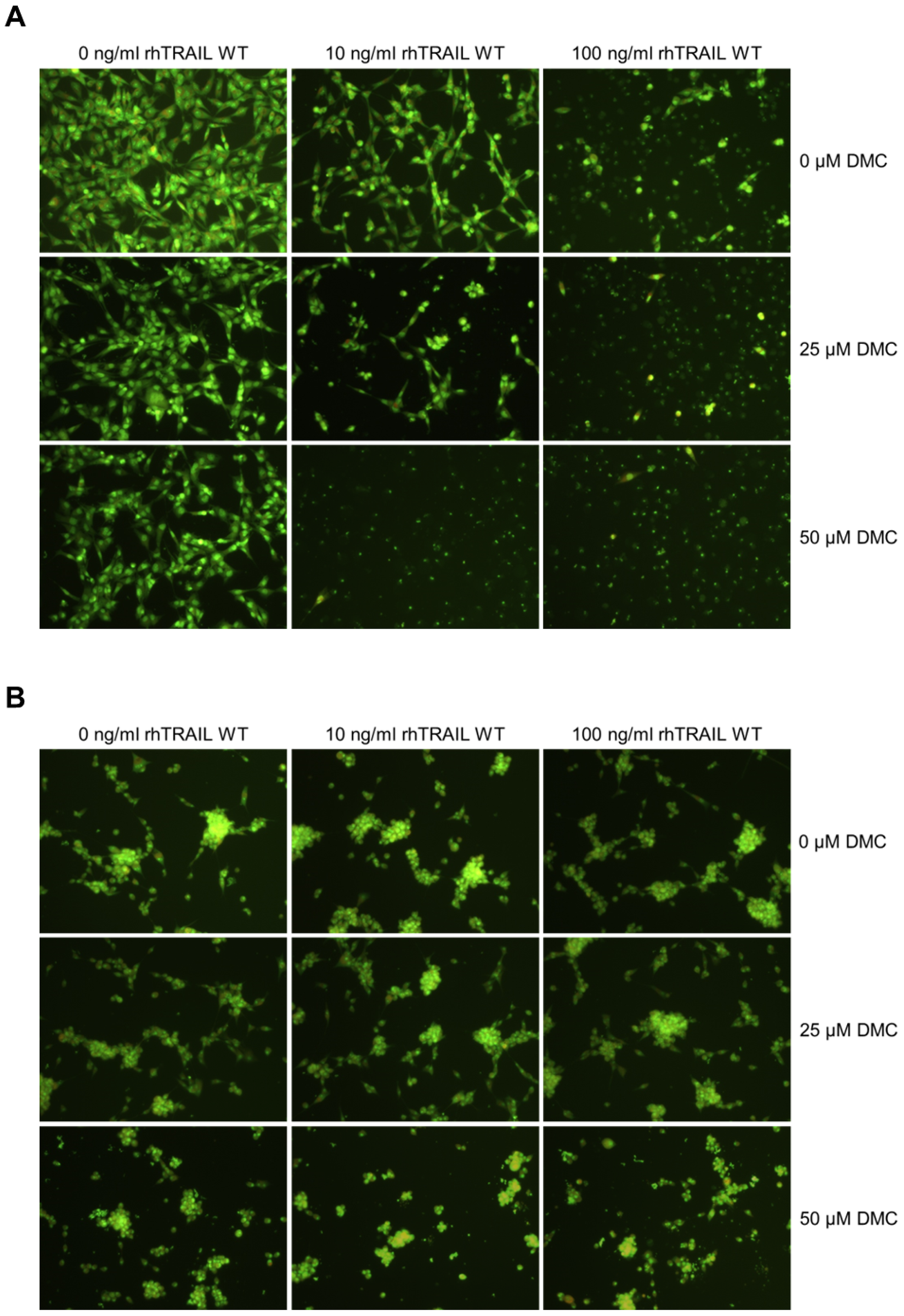

Supplement: Supplementary file 8 — Authors’ original file for figure 5 [file 40064_2014_1207_MOESM8_ESM.tiff]

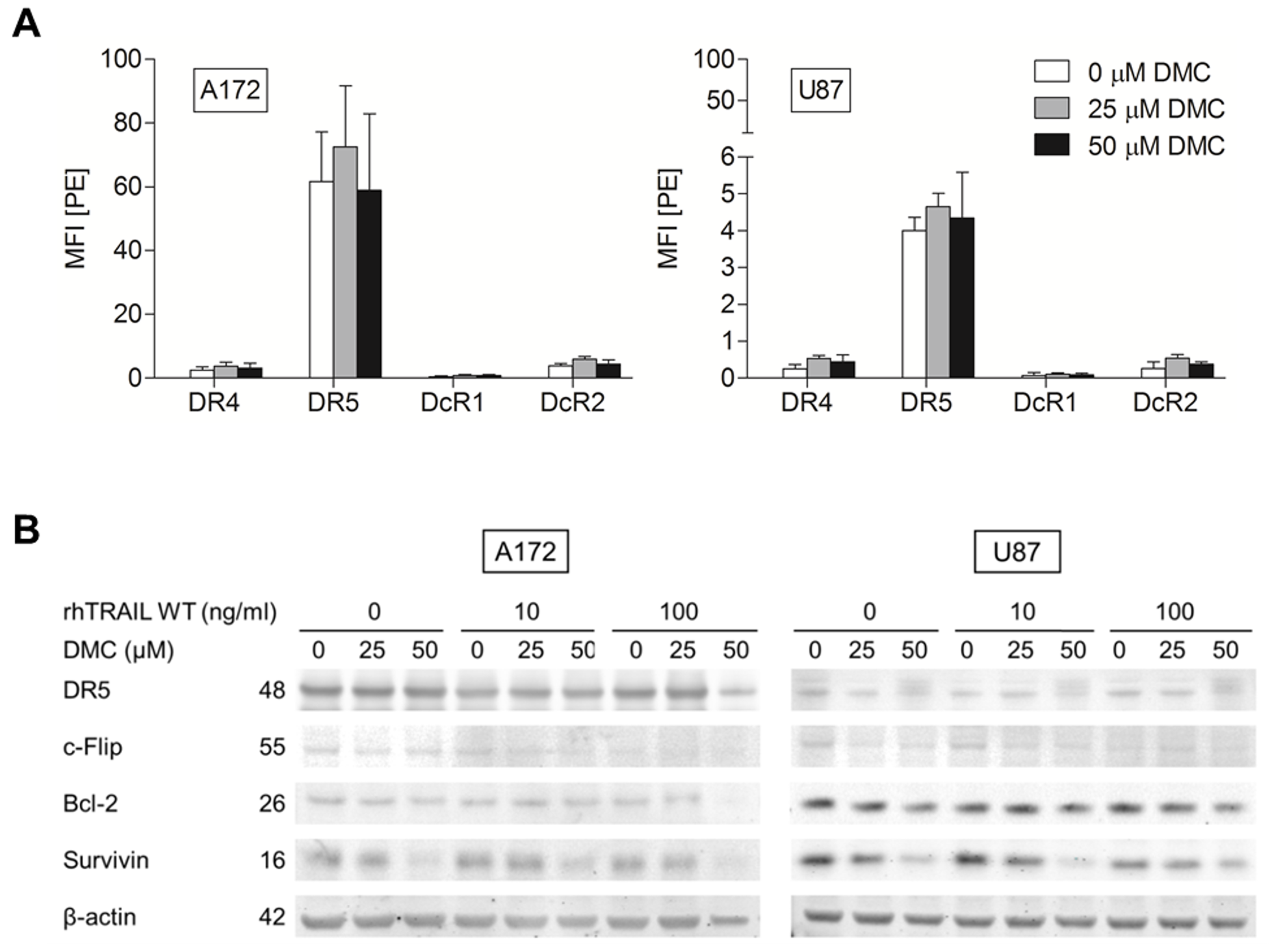

Supplement: Supplementary file 9 — Authors’ original file for figure 6 [file 40064_2014_1207_MOESM9_ESM.tiff]

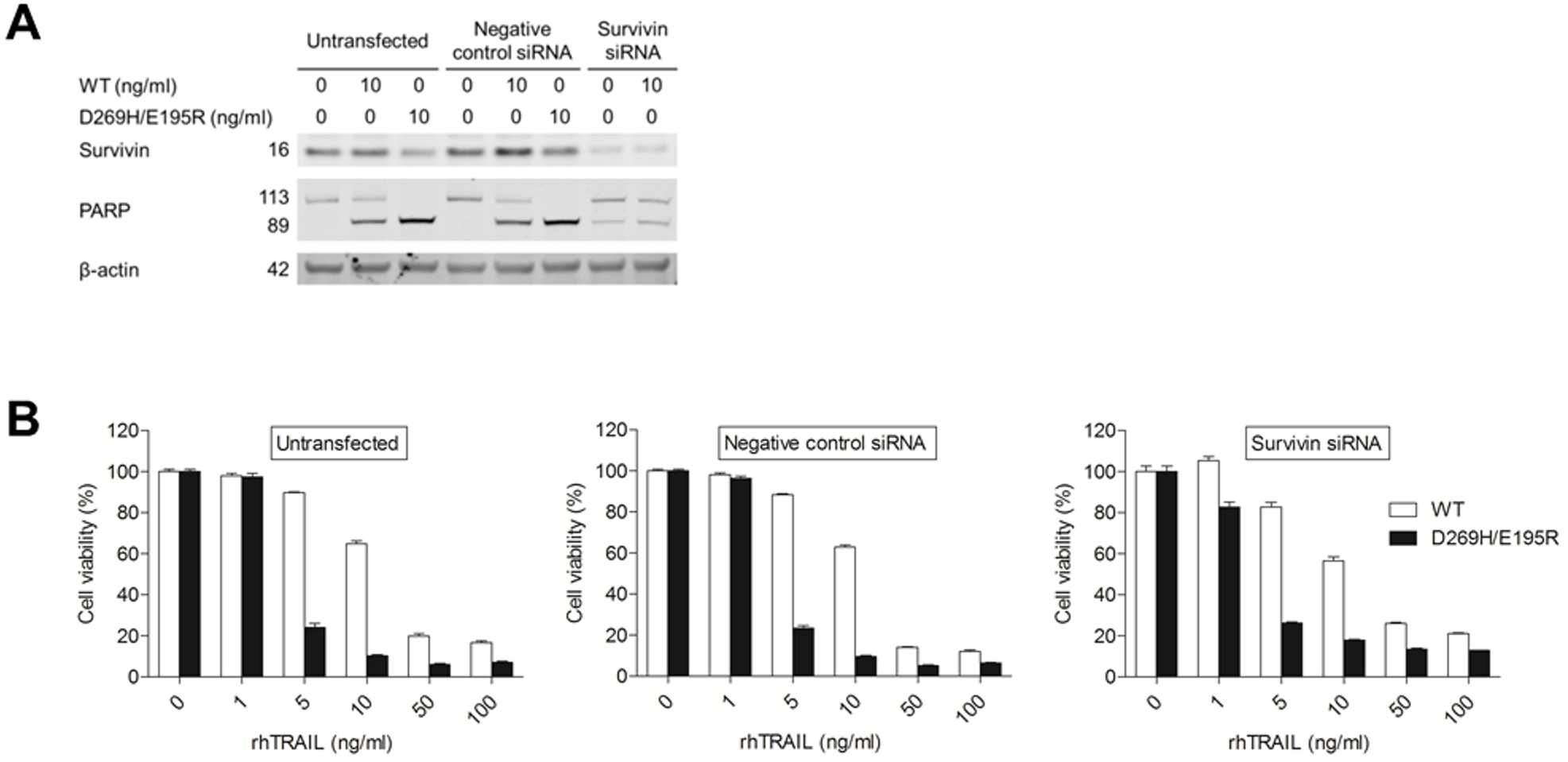

Supplement: Supplementary file 10 — Authors’ original file for figure 7 [file 40064_2014_1207_MOESM10_ESM.tiff]
